# Transplant renal vein thrombosis and stenosis were rescued by interventional radiology

**DOI:** 10.1002/iju5.12764

**Published:** 2024-07-27

**Authors:** Naoki Uchida, Keiichiro Miyajima, Takafumi Yanagiswa, Hirokazu Ashida, Mayuko Kawabe, Izumi Yamamoto, Takashi Yokoo, Takahiro Kimura, Fumihiko Urabe, Jun Miki

**Affiliations:** ^1^ Department of Urology The Jikei University School of Medicine Tokyo Japan; ^2^ Division of Nephrology and Hypertension, Department of Internal Medicine The Jikei University School of Medicine Tokyo Japan; ^3^ Department of Radiology The Jikei University School of Medicine Tokyo Japan

**Keywords:** interventional radiology, kidney transplantation, transplant renal vein thrombosis

## Abstract

**Introduction:**

Transplant renal vein thrombosis is a serious post‐transplant complication. We report a case in which a thrombus was found in the transplant renal vein and rescued the transplanted kidney utilizing interventional radiology.

**Case presentation:**

A 56‐year‐old woman underwent ABO‐compatible living donor renal transplantation due to impaired renal function caused by IgA nephropathy. On postoperative Day 13, there was a finding on transplant renal echocardiography that appeared to be an interruption of peripheral renal blood flow in diastole. Contrast‐enhanced computed tomography revealed that the vein was occluded due to the hematoma, and thrombosis was observed within, and distal contrast showed regurgitation into the collateral vessels. The stenosis was breached and balloon dilation restored progressive blood flow through interventional radiology.

**Conclusion:**

Although open surgical thrombectomy is mainly considered for treatment for transplant renal vein thrombosis, interventional radiology might be the alternative treatment option.

**Abbreviations & Acronyms**
TRVT: transplant renal vein thrombosis
IR: interventional radiology
HLA: human leukocyte antigen
GDUS: graft Doppler ultrasonography
CT: computed tomography

**Keynote message**

We performed removing venous thrombosis after kidney transplantation through interventional radiology such as balloon dilation.

## Introduction

TRVT has a dramatic clinical presentation and is one of the main causes of early graft disfunction after renal transplant, with a reported prevalence of 0.1%–4.2% of all transplant.^1^ Previous studies have reported that the treatment for venous thrombosis in allogenic renal grafts involves intravenous thrombolytic therapy and open surgical thrombectomy or allograft exploration.^2^, ^3^, ^4^ But there are no extensive randomized controlled studies have assessed the therapeutic risks and efficacy of different treatment options. Therefore, it is difficult to make a treatment decision.

Here, we report a case of venous thrombosis and venous stenosis after renal transplantation treated with transluminal ballon angioplasty, which avoided open surgical thrombectomy and transplant nephrectomy.

## Case presentation

A 56‐year‐old woman presented for transplant with a diagnosis of chronic kidney disease due to IgA nephropathy. The recipient and donor had no other comorbidities. The donor was the 57‐year‐old husband of the patient. HLA compatibility analysis revealed that the recipient's HLA type was A24, B48/54, DR4/14, while the donor's HLA type was A24/26, B44/52, DR13/15, resulting in a mismatch at 5 loci. The recipient had no donor‐specific antibody. The recipient's surgical method was performed by open surgery and the donor's was by laparoscopic transperitoneal approach.

**Fig. 1:**
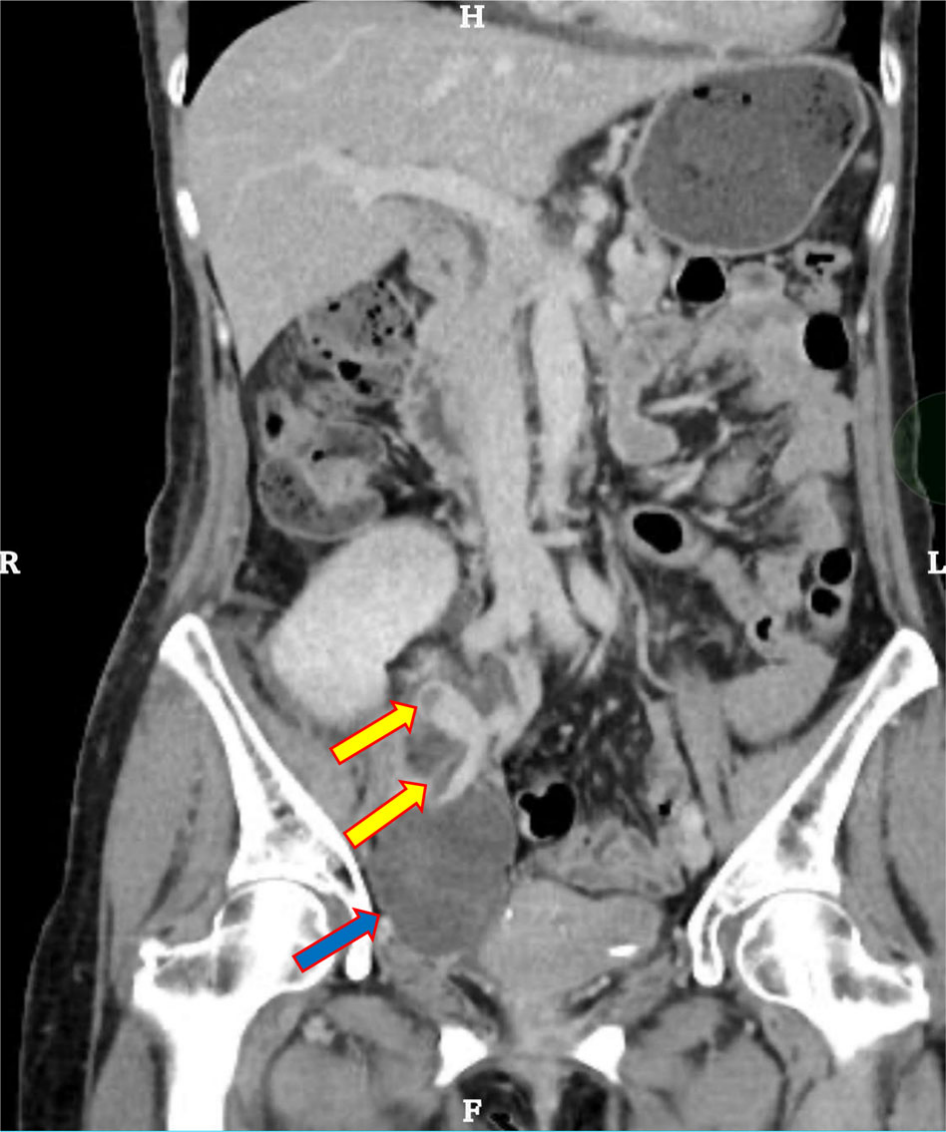
Thrombus in transplanted renal vein and right external iliac vein. Yellow arrows indicate the thrombus. Blue arrows indicate the hematoma.

The patient underwent an ABO‐compatible (O to O) living donor renal transplant in the right iliac fossa with the imposition of an end‐to‐side anastomosis between the kidney artery and the external iliac artery and between the renal vein and an external iliac vein. The renal vein and the external iliac anastomosis were performed with continuous suturing using 5‐0 Prolene, ensuring an adequate anastomotic diameter of the external iliac vein. After the surgery, GDUS showed good blood flow of the graft vessels with a vascular resistance index (RI) of 0.6–0.65, and no hydronephrosis or hematoma. We performed anticoagulation with heparin to maintain an APTT of 60–80 s. On postoperative Day 10, an exacerbation of the serum creatinine level (0.95 → 1.43 mg/dL), which had been improving with good progress, was observed. On postoperative Day 12 post‐transplantation, GDUS revealed stenosis of the external iliac vein and high pressure of backflow from distal to the stenosis. Contrast‐enhanced computed tomography revealed that the vein was occluded due to the hematoma, and thrombosis was observed within (Fig. 1).

**Fig. 2:**
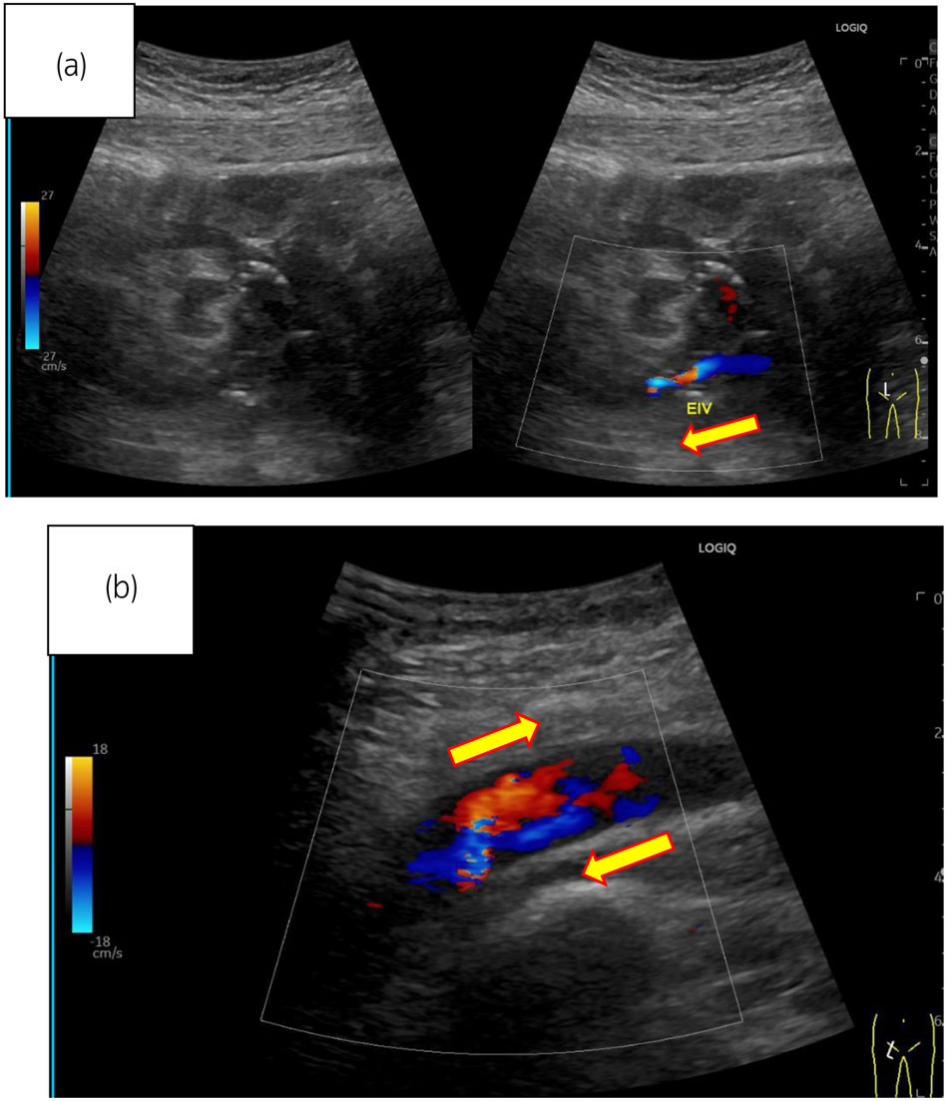
(a) The right iliac vein is refluxing near the anastomosis of the grafted renal vein. Yellow arrows indicate the blood flow. (b) The right external iliac vein is regurgitated by a thrombus. It merges with the prograde blood flow from the lower extremity, causing turbulent flow. Yellow arrows indicate the blood flow.

**Fig. 3:**
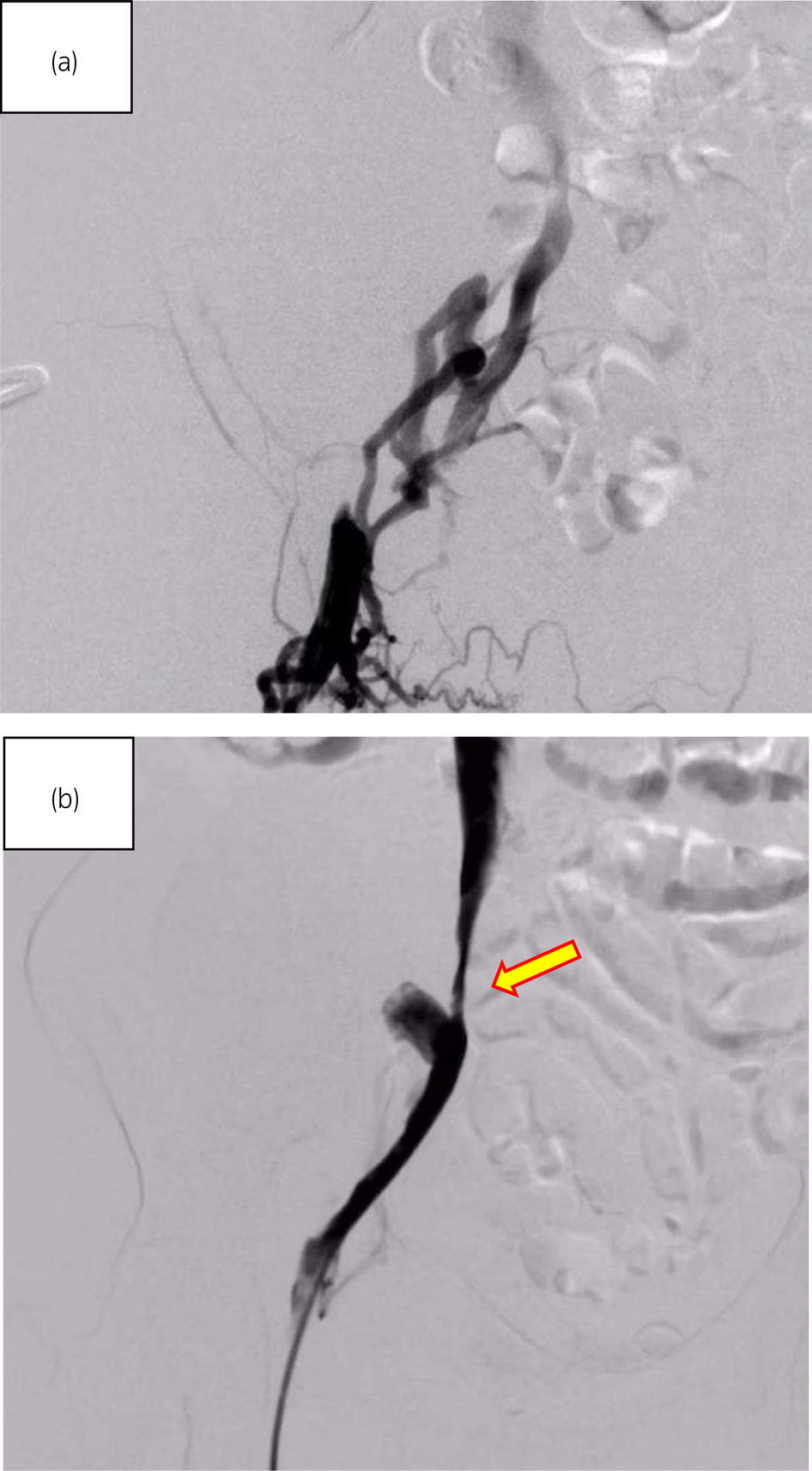
(a) The external iliac vein is occluded by venography from the femoral vein and perfusion is seen upstream via collateral channels. (b) Although the stenosis upstream of the anastomosis due to the hematoma remains (yellow arrows indicate the stenosis), the collateral vessels have disappeared and the flow is improved.

On postoperative Day 13, post‐transplantation, edema in the patient's right leg had appeared and GDUS revealed disruption of diastolic peripheral renal blood flow. The right iliac vein flows backward bordering the renal venous junction (Fig. 2a). The right common iliac vein merges with the prograde blood flow from the lower extremity, producing turbulent flow (Fig. 2b). The presence of a thrombus and impaired blood flow from the anastomosis of the transplant renal vein to the right external iliac vein led to the decision to treat the patient with IR. IR showed the external iliac vein is occluded by venography from the femoral vein and perfusion is seen upstream via collateral channels (Fig. 3a). Although the stenosis upstream of the anastomosis due to the hematoma remains, the collateral vessels have disappeared and the flow is improved (Fig. 3b). Guide wire passed through the stenosis and balloon dilation was performed using 6 and 7 mm percutaneous transluminal balloon catheter, which restored progressive blood flow. We did not perform thrombolysis and thrombectomy. The reason was that the thrombus volume was small and the preoperative CT showed a stenosis (almost occlusion) of the iliac vein near the anastomosis. After the intervention, follow‐up GDUS showed improvement in blood flow from the transplanted renal vein to the right iliac vein and CT revealed no enlargement of hematoma. Following the intervention, kidney function and symptoms gradually showed improvement. On Day 25 after transplantation, contrast‐enhanced CT confirmed the disappearance of thrombus, and the patient was discharged on Day 30 after the transplantation and experienced no other postoperative complications.

## Discussion

Shao‐Jie et al. previously reported that they treated TRVT utilizing pharmacomechanical thrombectomy plus transluminal ballon angioplasty which is well‐established for treating lower‐limb DVT.^5^ Compared to systematic thrombolysis, catheter‐contact thrombolysis is more direct and effective and reduces the risk of bleeding associated with high‐dose thrombolytic drugs. Additionally, open surgical thrombectomy is more invasive and increases the risk of anesthesia and infection.^1^ Therefore, it would be better to use percutaneous intravenous thrombectomy for some selected cases. In our case, the presence of a thrombus was suspected on contrast‐enhanced CT, and thanks to the onsite availability of a specialist interventional radiologist, the location of the thrombus could be precisely identified through IR. These enabled the selection of the treatment method.

One of the discussion points of our case would be that we did not place an inferior vena cava (IVC) filter before the intravenous thrombectomy, which might have caused some risks to occur the pulmonary embolism. However, the small volume of the thrombus, coupled with the use of anticoagulant medication, led to the determination that the risk of embolism was low during the IR. On the other hand, a previous report showed that among 70 patients who underwent retrievable IVC filter placement to prevent thrombus dislodgement during catheter‐directed thrombectomy for DVT, the thrombus was dislodged and trapped by the IVC filter in 22 patients (31.4%).^7^ Thus, we had better to discuss the necessity of IR filter before the induction of the IR, and considering the patient's safety and the results of the previous study,^7^ it may have been prudent to place an IVC filter as a precaution.

In the present case, we rescued the transplanted kidney by utilizing IR. Edema and findings of renal vein stenosis appeared first on the 13th day post‐transplantation, which was earlier than in a previous study.^5^ In general, the causes of TRVT are diverse, including surgical technical problems, immunosuppressive factors, and donor and recipient factors.^6^ In this case, it is presumed that a post‐operative hematoma around the anastomosis caused stasis of the transplant renal venous return and thrombus formation, leading to embolization and subsequent occlusion. If there were complete occlusion, it could lead to congestive kidney and irreversible renal function loss, resulting in nephrectomy. However, in this case, contrast imaging revealed the presence of collateral pathways, suggesting that there was still some blood circulation, albeit in small amounts.

In conclusion, percutaneous intravenous thrombectomy combined with balloon angioplasty could be a treatment option for TRVT. As the anastomotic hematoma showed a tendency to improve over time, we determined that hematoma removal was not imperative. Ideally, laparotomy (hematoma removal and thrombectomy) should be performed. However, this process involves time, including pre‐operative investigations, before laparotomy can be scheduled. In our hospital, endovascular treatment was more readily accessible; therefore, it was performed first, resulting in a successful outcome. This report does not exclude the possibility of open hematoma removal but introduces an additional option. Yet, conducting thrombectomy without an IVC filter may pose risks. While the treatment successfully saved the transplanted kidney in this case, more research is needed to ensure its effectiveness and safety. We hope this method will become a viable option for TRVT treatment in clinical settings.

## Author contributions

Naoki Uchida: Conceptualization; project administration; writing – original draft. Keiichiro Miyajima: Conceptualization; methodology; writing – original draft. Takafumi Yanagiswa: Methodology; project administration; writing – original draft. Hirokazu Ashida: Conceptualization; project administration; writing – original draft; writing – review and editing. Mayuko Kawabe: Conceptualization; project administration; writing – original draft; writing – review and editing. Izumi Yamamoto: Conceptualization; project administration; writing – review and editing. Takashi Yokoo: Writing – review and editing. Takahiro Kimura: Writing – review and editing. Fumihiko Urabe: Conceptualization; data curation; investigation; methodology; project administration; visualization; writing – original draft; writing – review and editing. Jun Miki: Conceptualization; project administration; writing – review and editing.

## Conflict of interest

The authors declare no conflict of interest.

## Approval of the research protocol by an Institutional Reviewer Board

Not applicable.

## Informed consent

Consent to participate and for publication was acquired from the patient.

## Registry and the Registration No. of the study/trial

Not applicable.
